# Common Neurologic Diseases in Geriatric Dogs

**DOI:** 10.3390/ani14121753

**Published:** 2024-06-10

**Authors:** Luciano Espino, Natalia Miño

**Affiliations:** Departamento de Anatomía, Producción Animal y Ciencias Clínicas Veterinarias, Facultad de Veterinaria de Lugo, Universidad de Santiago de Compostela, 27002 Lugo, Spain; natalia.mino@usc.es

**Keywords:** central nervous system, spinal cord diseases, brain diseases

## Abstract

**Simple Summary:**

The geriatric population of dogs represents a high percentage of the patients in veterinary practice. As with other organ systems, degenerative, neoplastic, and vascular diseases are most prevalent in older dogs. This review will summarize the clinical presentation, diagnosis, and therapeutic options of the more frequent diseases affecting the central nervous system of geriatric dogs.

**Abstract:**

The increase in the canine geriatric population means that veterinarians are more often confronted with diseases that are more prevalent in patients in this age group. As in other organ systems, degenerative, neoplastic, and vascular diseases are the most prevalent neurologic disorders in older dogs. A neurological disease in an older dog poses a challenge for the clinician due to the presence of concomitant diseases and age-related changes that make it difficult to interpret the neurological examination. In addition, given the age of the patients, some owners do not allow advanced imaging tests, and it is necessary to establish the most likely presumptive diagnosis to initiate treatment. Although many of these diseases can cause clinical signs that can be very upsetting, some of them can be managed with symptomatic therapy and have a good prognosis, such as idiopathic vestibular syndrome. Moreover, advances in and the greater availability of therapeutic options such as surgery and radiation therapy may increase survival and quality of life in diseases with a more serious prognosis, such as tumours. The aim of this review is to summarize the clinical presentation, diagnosis, and treatment of the more frequent diseases affecting the central nervous systems of geriatric dogs.

## 1. Introduction

Older pets represent more than 40% of patients in general practice, and this proportion is likely to increase in the future as dogs live longer, a fact that should encourage veterinary clinicians to expand their focus on senior pets [1]. Aging is the single most important risk factor for a wide variety of diseases, including degenerative, neoplastic, and vascular illnesses [2]. The nervous system is not immune to aging, and degenerative, neoplastic, and vascular diseases are the most common neurologic disorders in older dogs [3,4]. A geriatric dog with a nervous system disease is always a diagnostic challenge for the clinician. Some deficiencies associated with age (e.g., the absence of a patellar reflex) and the presence of concurrent medical issues (especially orthopaedic conditions) can interfere with the interpretation of neurological examinations and neurolocalization [5]. Moreover, there may be owners who do not wish to pursue advanced diagnostic tests for several reasons, including the age of the pet, concern and fear regarding certain test procedures or outcomes, and financial constraints. Among the general causes of neurological diseases in older dogs, in this revision we focus on the most prevalent brain and spinal cord diseases. Within degenerative diseases of the brain, canine cognitive dysfunction has a high prevalence in older dogs; readers can find an in-depth description in other publications and in a review article in this special issue [2,3,4]. Many of these diseases can cause clinical signs that can be very upsetting to owners, but some of them can be treated and palliated successfully, and owners should be cautioned not to rush into making decisions primarily based on the initial clinical appearance [4]. Due to these limitations, the aim of this review is to summarize the clinical presentation, diagnosis, and treatment of the more frequent diseases affecting the central nervous systems of geriatric dogs, with particular attention to those clinical data that could help establish a more accurate presumptive diagnosis in cases in which advanced diagnostic tests are not available.

## 2. Brain Diseases

### 2.1. Intracranial Neoplasia

Intracranial neoplasia is a major cause of morbidity and mortality in dogs, with primary brain tumours representing 2–5% of all cancers [6]. Although accurate data for the true incidence of intracranial neoplasia in dogs is limited, it has been reported in approximately 2–4.5% of dogs. These values are like those described in human patients, presenting a similar incidence of primary and secondary neoplasms [7,8,9,10]. In this review, we are going to focus on the most important data on the epidemiology, diagnosis, and treatment of the most common primary intracranial tumours encountered in clinical practice in dogs: meningiomas and gliomas [7,9]. 

Most of these brain neoplasms occur in middle-aged to older dogs (median age: 8 years). Although they can occur in any breed, gliomas are overrepresented in brachycephalic breeds. Almost 80% of gliomas occur in these breeds, while meningiomas are more common in larger breeds of dogs [11,12,13]. There is not a clear sex predisposition, although gliomas tend to be more common in male dogs [12,14]. They are prone to occur as solitary mass lesions, and tumours involving forebrain structures are more frequent than those in the brainstem [12,13,15]. 

Intracranial neoplasms cause clinical signs of brain dysfunction by directly invading or compressing brain tissue and, secondarily, by causing peritumoral oedema, neuroinflammation, obstructive hydrocephalus, and intracranial haemorrhage [6]. Seizures and neurological deficits indicative of a focal forebrain lesion are the most common clinical presentations of brain neoplasia [7,12,13,15]. However, neoplasia in the fronto-olfactory region is often associated with only historical evidence of brain disease, such as seizures or behavioural changes, and a normal interictal neurological examination. New-onset seizures might represent an early warning sign for the presence of a brain tumour, as other more subtle signs of neurologic disease in dogs might go unnoticed by their owners [12].

**Figure 1:**
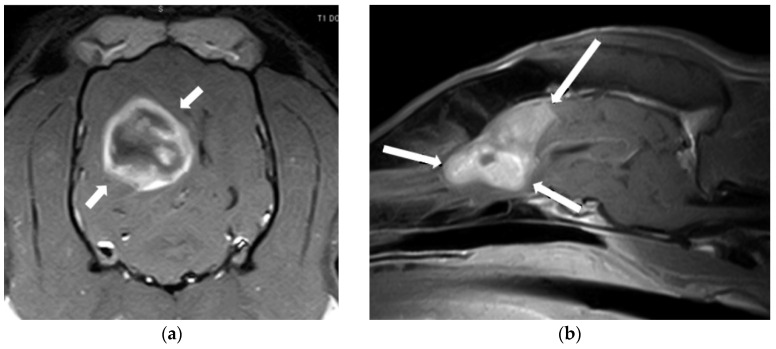
MRI images of an intracranial glioma (**a**) and a meningioma (**b**). (**a**) Dorsal, post-contrast T1W image illustrating a heterogeneously hypointense and ring-enhancing intra-axial mass in the right parieto-temporal lobes (arrows). (**b**) Sagittal, post-contrast T1W image of a well-demarcated, extra-axial mass in the region of the olfactory bulb and frontal lobe, showing a homogeneous and strong contrast enhancement.

Regarding diagnosis, a complete blood count, chemistry profile, and urinalysis are generally indicated to evaluate the animal’s systemic health status. As brain neoplasms are more frequent in older dogs, concurrent comorbidities on thoracic or abdominal imaging can be detected in some patients. Metastatic disease is very uncommon in primary brain tumours, and the comorbidities found rarely affect the diagnostic and therapeutic recommendations [12,13,16]. Cross-sectional diagnostic imaging techniques are required for a presumptive diagnosis, with magnetic imaging resonance (MRI) being the preferred modality, as in other intracranial diseases (Figure 1).

The reported sensitivities of MRI to correctly identify meningiomas and gliomas range between 60 and 100% and between 58.8 and 68.8%, respectively [12,17,18]. The significant overlap that exists in the imaging features of gliomas, cerebrovascular accidents, and inflammatory lesions, and between meningiomas and histiocytic sarcomas and granular cell tumours, may result in the misdiagnosis of these categories of diseases [18,19,20,21]. Cerebrospinal fluid (CSF) analysis is rarely diagnostic because most of the detected abnormalities are nonspecific, and neoplastic cells are detected very infrequently [6,12,22]. Obtaining CSF from dogs with brain neoplasia and intracranial hypertension carries a risk of clinical deterioration. Although this risk is low, it should be critically assessed in each patient and evaluated in the context of the likelihood of obtaining a non-specific test result. Advanced imaging of the brain should always precede CSF collection to best evaluate individual patient risk [6]. The definitive diagnosis of intracranial tumours is based on the histopathologic assessment of representative tissue. In dogs with brain lesions that cannot be safely or practically sampled using excisional biopsy, stereotactic brain biopsy (SBB) can be used to obtain brain tissue for definitive diagnosis. Although there is still limited information about SBB in veterinary medicine, it can provide sufficient information to guide therapeutic decisions with a good diagnostic accuracy and without severe adverse events [23,24].

The paucity of data and controversy around the most appropriate therapy for intracranial tumours make it difficult for clinicians to confidently advise owners on treatment decisions, especially with the combination of different therapeutic modalities. Symptomatic and definitive therapy (surgery, chemotherapy, radiotherapy, or a combination of these) are the two options for the treatment of brain neoplasia [6,11,22]. The primary goal of symptomatic treatment is to improve the quality of life of patients and their caregivers. Corticosteroids targeting peritumoral oedema and antiepileptic drugs for tumour-associated structural epilepsy form the mainstay of palliative care in these patients [6,11,22]. Hyperesthesia has been reported infrequently in dogs with brain neoplasia and can be respond to with corticosteroids alone or in combination with analgesics [8,25]. In patients with tumours causing secondary obstructive hydrocephalus, surgical CSF diversion via the placement of an intraventricular shunt is an effective method of alleviating the clinical signs of intracranial hypertension [26,27].

Despite the marked variability in treatment modalities (surgery, radiation therapy, chemotherapy), the median survival time (MST) for definitively treated cases (mean among reports: more than 300 days) is longer than for symptomatically treated cases (mean among reports: 65 days), suggesting that definitive therapies provide a significant survival benefit to dogs with intracranial tumours. However, the benefit is different depending on the treatment modality and the type of neoplasm [6,11,12,13,22]:

- Chemotherapy: its use is limited due to the presence of the blood–brain barrier (BBB), which reduces the number of drugs than can access the central nervous system. The most commonly used chemotherapeutics for brain tumours are the alkylating agents lomustine (CCNU), carmustine (BCNU), and temozolomide (TMZ) for gliomas, and the antimetabolite hydroxyurea in the case of meningiomas [6,11,13,22,28]. Despite the weak evidence to support their efficacy in the treatment of canine intracranial neoplasia, it seems as though most reported chemotherapeutics, either alone or combined with other anticancer therapies, could have some beneficial effect on survival [12,13,29,30].
- Surgery: most published information with meaningful case numbers is related to more easily accessible canine meningiomas [11,13,20]. In a recent study, the median survival time of dogs with meningiomas treated with surgical resection was 386 days [13]. Surgical treatment of gliomas is infrequently attempted as approaching and removing them is technically demanding due to their intra-axial location [6,28].
- Radiation therapy (RT): it has been shown to be beneficial for the treatment of intracranial tumours used as a monotherapy or adjunctive modality [11,12,13,22,31,32,33]. Although meningiomas are associated in the literature with a significantly better prognosis in terms of survival compared to gliomas, recent studies reported similar survival times in both types of tumours [33,34]. Another important beneficial effect of radiation therapy is the increase of seizure freedom. This can be crucial because the recurrence of seizures is a common reason for euthanasia in these patients [31,33,35].

### 2.2. Cerebrovascular Disease

In humans, cerebrovascular disease (CVD) is one of the most common causes of death worldwide [36]. Information about the prevalence of CVD in dogs is limited, but it is suspected to be considerably lower than in humans [37]. Cerebrovascular disease refers to any abnormality of the brain resulting from a pathological process of the supplying blood vessels, such as thrombosis or embolism (ischemic strokes) or haemorrhage (haemorrhagic strokes), leading to temporary or permanent damage to the brain [38]. A stroke, or cerebrovascular accident, is defined as the sudden or abrupt onset of focal neurological deficits resulting from an intracranial vascular event and, by convention, with clinical signs lasting at least 24 h [39]. A stroke is the most common clinical manifestation of cerebrovascular disease in dogs [37,40,41,42,43,44,45,46]. An additional form of cerebrovascular ischemia is transient ischemic attack (TIA), which can be defined as a symptomatic episode of brief, focal neurologic deficit secondary to embolism, vascular constriction, or spasm that resolves within 24 h [47]. Strokes are more frequently diagnosed in older dogs (8 years or more), but they have no clear sex predisposition [45,48,49,50]. In one study, Cavalier King Charles Spaniels and Greyhounds were overrepresented [40]. 

The rapid onset of neurological deficits is the most characteristic sign of a stroke. Ischemic strokes may be nonprogressive or have limited progression if secondary vasogenic oedema or haemorrhagic conversion develops. Haemorrhagic strokes may have a slightly more gradual onset and longer progression [37,42,46]. The neurological deficits are often focal and related to the localization and extent of the lesion [40]. The relative anatomic incidence of CVD has been variably reported, with the forebrain (middle cerebral artery), thalamus (perforating arteries), and cerebellum (rostral cerebellar arteries) being the most affected regions [40,45,48,49,50]. 

**Figure 2:**
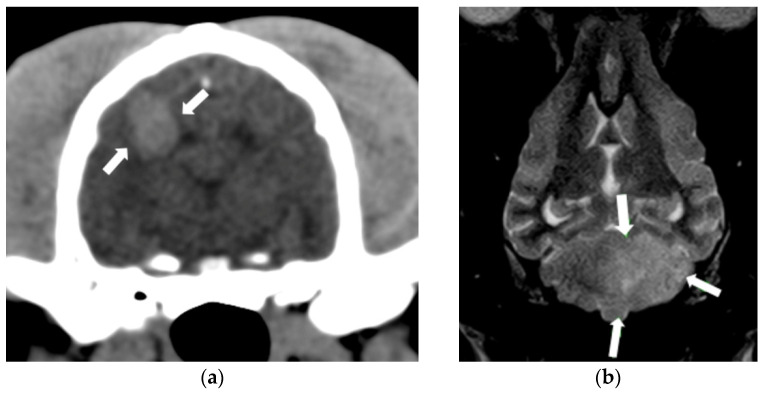
(**a**) Transverse CT of the head in the mid cerebrum. A hyperdense lesion is present in the region of the right parietal lobe, causing a mild compression of the right lateral ventricle (arrows). This lesion represents haemorrhagic infarction. (**b**) T2-weighted dorsal image. A large, sharply-demarcated, hyperintense lesion is present in the region of the left rostral cerebellar artery (arrows). This lesion is consistent with an ischemic infarct.

A tentative diagnosis of CVD is based on clinical signs, neurological findings, and the results of advanced brain imaging. Baseline diagnostic tests for dogs with suspected cerebrovascular disease should include complete blood cell counts, full serum biochemistry, urinalysis, blood pressure measurements, and the assessment of adrenal and thyroid function to rule out an underling cause of the stroke (kidney disease, hyperadrenocorticism, hypothyroidism, or hypertension). However, in almost half of cases, a concurrent medical condition is not identified [37,40,45,48]. Advanced brain imaging is indicated to rule out other causes of neurologic signs and to define the extent of the affected area. MRI is superior to computed tomography (CT) for the detection of ischemia due to its excellent soft tissue contrast and is the imaging diagnosis technique recommended for the presumptive diagnosis of CVD in dogs [42,46,51]. Computed tomography (CT) is particularly sensitive for detecting acute haemorrhage, which appears as a homogeneously hyperdense image (Figure 2) [37,51]. The MRI findings of ischemic stroke include well-defined, sharply-demarcated lesions with minimal to no mass effect that are limited to the vascular territory (Figure 2) [42,45,51].

CSF analysis may help rule out inflammatory disease, and it can be normal or show nonspecific changes [42,45,46,48]. Once the diagnosis of a stroke is made, treatment is mainly supportive, focused on minimizing secondary damage or complications and identifying and treating any potential underlying cause. The goals of treatment are to maintain good cerebral perfusion pressure and to supplement oxygen. Fibrinolytic therapy has not been evaluated in cases of acute ischemic strokes in dogs because few canine patients that undergo a stroke are evaluated in the same acute time frame as humans [42,46,51]. Prognosis for recovery depends on the severity and location of the lesion [40,45,49]. The presence of concurrent medical conditions has been associated with shorter survival times [40,45,49]. However, in a recent study, there was no association between the outcome and the nature of concurrent diseases [48]. Recovery within weeks with only supportive care is commonly reported in dogs with ischemic stroke [40,45,49]. Limited information about recurrence has been published, but it can more likely occur in dogs with an identifiable medical condition [40,48,50].

### 2.3. Idiopathic Vestibular Syndrome

Vestibular dysfunction is relatively common in geriatric dogs, with an overall 0.36% reported in primary veterinary care [52]. There are several conditions that may cause vestibular signs. However, in older dogs, we can narrow the list of differentials and focus on the three most common diagnoses (idiopathic, cerebrovascular disease, and brain neoplasia) [53,54]. Idiopathic vestibular syndrome (IVS) can be characterized as an acute or peracute, improving, non-painful peripheral vestibular disorder that often affects geriatric dogs [55,56]. The aetiology of this disease remains undetermined. In humans, there are several well-described causes of acute vestibular syndrome: Ménière’s disease, benign paroxysmal vertigo, and acute vestibular neuritis [57,58]. It seems likely that dogs diagnosed with IVS may in fact have different underlying causes for these signs, therefore explaining the different presentations (acute or chronic, progression or not of clinical signs, and concurrent facial nerve deficits or not), MRI findings (enhancement or not of the cranial nerves VII and/or VIII, atrophy of the utricle, lack of suppression of the inner ear), outcomes, and recurrence of clinical signs [53,56,59,60]. 

The clinical signs of vestibular dysfunction are usually unilateral and commonly include loss of balance, asymmetric rolling, leaning, or falling ataxia, head tilt, spontaneous nystagmus, and/or strabismus. Moreover, nausea and emesis can be common manifestations of acute vestibular system dysfunction in dogs [61]. IVS is diagnosed by exclusion of the other causes of peripheral vestibular disease, and diagnosis is presumptive in many cases. Some clinical variables associated with idiopathic vestibular syndrome include higher age, large breed, improving clinical signs, pathological nystagmus, facial nerve paresis, the absence of Horner’s syndrome, and a peripheral localization [53].

Regarding diagnosis, a detailed neurological examination and comprehensive blood tests, including thyroid values, blood pressure, and otoscopic examination, remain crucial. A through workup may also involve MRI and CSF to rule out other causes, but they are usually reserved for patients that present other neurological deficits or do not improve with supportive treatment.

Symptomatic therapy includes fluid therapy and antiemetics. Maropitant, metoclopramide, and ondansetron are effective at reducing vomiting in dogs, but only ondansetron has been demonstrated to significantly reduce the signs of nausea associated with vestibular syndrome [62,63]. In human medicine, antihistaminergic drugs, mainly betahistidine, are frequently prescribed for the management of acute vestibular syndrome. However, currently, there are limited data supporting the use of these drugs in dogs with IVS [64]. In human patients with benign positional paroxysmal vertigo, positional exercises are used to try to reposition the otoliths out of the canals. There are several limitations to recommending its use in veterinary medicine because it is not proven that IVS shares an aetiology with BPPB in humans, and there is only one study that reported positive outcomes in a limited number of dogs with IVS treated with positional exercises [65]. 

Most cases show improvement within a couple of days, and complete resolution of clinical signs is typically observed within 2–4 weeks. Mild residual clinical signs, such as a slight head tilt or mild ataxia, can persist in some cases for life [55]. If this clinical course is not followed, imaging with MRI or CT is recommended [55,56]. In one recent study, in 19 out of 85 dogs (22%), there was a recurrence of the clinical signs at least once over the following 12 months [56]. Incomplete recovery was most frequently seen in patients presenting with cranial nerve enhancement on MRI. However, a history of previous episodes of vestibular dysfunction was associated with an increased chance of the resolution of clinical signs [56]. 

A summary of the main data on the clinical presentation, diagnosis, and treatment of intracranial diseases is included as Table S1 in the Supplementary Materials.

## 3. Spinal cord Diseases

### 3.1. Intervertebral Disc Disease

Intervertebral disc disease is the most common spinal disorder in dogs [66,67]. Two types of degenerative intervertebral disc disease have historically been recognized: Hansen type I intervertebral disc disease, or intervertebral disc extrusion (IVDE), and Hansen type II intervertebral disc disease, or intervertebral disc protrusion (IVDP). IVDE is most recognized in middle-aged, chondrodystrophic, small-breed dogs, while IVDP more commonly affects older, non-chondrodystrophic, large-breed dogs [68,69,70,71]. Although acute disc extrusions do occur, they are not frequent in older dogs, and we are going to focus this review on intervertebral disc protrusions, which are more prevalent in geriatric dogs [69,71,72,73,74,75,76].

Intervertebral disc protrusion develops most commonly in older, large, non-chondrodystrophic dogs and has no reported sex predisposition; the thoracolumbar spine is the most affected area [69,71,72,73,74,75,76,77]. Affected dogs typically demonstrate the chronic onset of progressive, but relatively mild, clinical signs, such as proprioceptive ataxia and ambulatory paresis. IVDP is not usually very painful. Nevertheless, pain may be present depending on the existence of nerve root compression [68,69,71,72,73,74,75,76,77].

Although intervertebral disk protrusions can be diagnosed by a variety of imaging modalities, MRI is currently considered the imaging modality of choice [72,74]. Radiographic abnormalities described in dogs with IVD protrusions are unspecific and include vertebral end-plate sclerosis, spondylosis deformans, and narrowing of the intervertebral disc space [69,78]. MRI allows the assessment of spinal cord parenchyma and the detection of spinal cord signal changes, which have prognostic value. In cases with multiple sites of spinal cord compression, the detection of hyperintensity on T2-weighted images can identify the site with the worst compression [73,74,79]. However, it is not always easy to differentiate between intervertebral disc extrusion and protrusion, and one study suggests that although MRI proposed guidelines can aid in differentiating between these two entities, they cannot replace thorough clinical training and experience [74]. 

As for IVD extrusion, both medical and surgical treatment options exist for the management of IVD protrusion. However, very little information is available about the results of these therapeutic options [69,72,75,76,79]. Medical treatment consists of combinations of relative rest, physical rehabilitation, and the administration of analgesics, muscle relaxants, and anti-inflammatory drugs [69,72,80,81]. Despite the limited available data, medical treatment tends to have a lower success ratio and worse long-term outcome than surgery. New therapeutic strategies, such as medical ozone or perineural glucocorticoid injection, could be considered as an adjuvant treatment in dogs that do not show an adequate response to conventional medical treatment [77,82].

**Figure 3:**
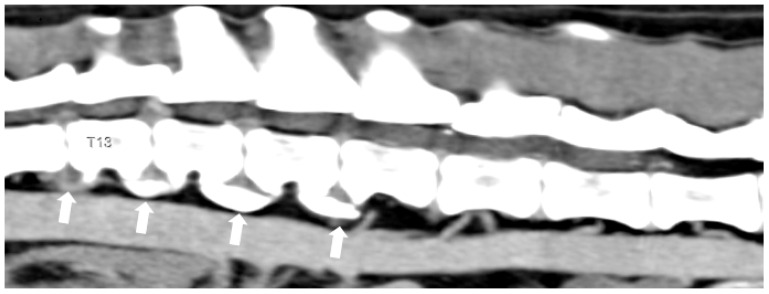
Midsagittal reconstructed CT scan of a dog with thoracolumbar myelopathy, revealing evidence of several intervertebral disc protrusions, narrowing of the affected intervertebral spaces, and spondylosis (arrows). Spinal cord compression is marked at T13-L1, but there are also other less severe protrusions at T12-T13, L1-L2, and L2-L3.

Intervertebral disc protrusion presents several problems for decompressive surgery decision-making and the management of client expectations. One problem is deciding on which disc to operate because affected dogs commonly have more than one protrusion associated with evidence of spinal cord injury (Figure 3) [69,72,79]. Another limitation is that the affected disc cannot be removed with conventional surgical techniques used in extruded discs, and the recommended surgeries (lateral corpectomy or hemilaminectomy with annulectomy) are technically more demanding [69,72,75,76,79]. Moreover, affected dogs are also often susceptible to other diseases, notably degenerative myelopathy [83], and some patients have other comorbidities that can increase the surgical and anaesthetic risks [79,82]. Finally, chronic spinal cord compression may result in cord atrophy with significant irreversible axonal supporting tissue and vascular injury, and a worsening of neurological dysfunction following decompressive surgery is not uncommon [69,72,75,76,79]. Despite these limitations, surgical treatment is associated with a higher success rate and longer survival.

### 3.2. Degenerative Myelopathy

Degenerative myelopathy (DM) is one of the most common chronic diseases of the spinal cord in dogs and has been known for more than 50 years [84]. The overall prevalence of the disease among all dogs is estimated at 0.19%, although due to its genetic origin, the prevalence varies widely among breeds and countries [85,86,87,88]. DM is an adult-onset (usually older than 8 years at the onset of clinical signs) chronic, progressive neurodegenerative disease with an autosomal recessive with incomplete penetrance mode of inheritance (OMIA 000263-9615), occurring primarily in large-breed dogs [87]. It was initially described in German Shepherds but has now been reported in several pure- and mixed-breed dogs such as Pembroke Welsh Corgis, Boxers, Rhodesian Ridgebacks, Collies, and Bernese Mountain Dogs [84,89,90]. DM is associated with mutations in the gene encoding Cu/Zn superoxide dismutase 1 (SOD1), one of the most abundant proteins in the central nervous system that functions as a free radical scavenger [90,91]. DM is considered a spontaneous model of familial amyotrophic lateral sclerosis because around 20% of patients with this disease present similar mutations in the SOD1 gene [92].

Based on histopathology, DM can be best described as a multisystem central and peripheral axonopathy [83]. In general, the spinal cord pathology of DM is consistent with noninflammatory axonal degeneration. All white matter folliculus are affected, with predominance in the lateral and dorsal folliculus, and the lesions involve proprioceptive pathways as well as the upper motor neuron tracts [93,94]. The pathological changes are more severe in the thoracic spinal cord segments, and there is moderate degeneration in the cervical and lumbar segments [93]. This pattern of distribution of the lesions explains the clinical signs of the disease. 

Canine DM is characterized by a slowly progressive, often asymmetric, general proprioceptive ataxia and upper motor neuron spastic paresis of the pelvic limbs. DM is a non-painful disease beginning in late adulthood (most dogs are at least 8 years of age at the onset of clinical signs) and is without sex predisposition [83]. The clinical signs will progress and ascend to affect the thoracic limbs, urinary and faecal control, brainstem, and respiratory muscles [85,95,96,97,98]. Death results from respiratory dysfunction, although most dogs are euthanized before reaching this point [83,97,98]. Clinical signs and progression are relatively uniform among dogs of the same breed. However, various factors, such as the canine SP110 gene found as a modifier in Pembroke Welsh Corgis, can increase the probability of developing DM and earlier onset of disease [99].

Definitive diagnosis is determined postmortem on histopathologic examination of the spinal cord, and, therefore, antemortem diagnosis remains presumptive after the exclusion of other common chronic myelopathies and myasthenia gravis [83]. An accurate presumptive diagnosis of DM should be made based on a lack of clinically relevant compressive myelopathy on MRI, an absence of anomalies on the CSF analysis, and the genetic test for SOD1 mutation [83,98]. It is important to remember that the SOD1 testing is inadequate as a sole diagnostic tool because of incomplete penetrance of the mutation, and, therefore, this test only identifies dogs at risk of developing DM. Moreover, there are two scenarios in which it is difficult to achieve a presumptive diagnosis. One is because, in some cases, owners refuse to pursue MRI and CSF because of anaesthesia and costs. The other is due to the coexistence of other myelopathies that can confound the diagnosis, for example, mild disc protrusions [83,98]. Some studies have found potential biomarkers that could play a role in improving the diagnosis and monitoring of DM, but they are not used in clinical practice [94,100,101].

There is no therapy for degenerative myelopathy, and most of the treatment regimens have been empiric, with a lack of evidence-based medicine. Several researchers have evaluated different medical therapies, and most of them found no beneficial effects [83]. In one study, dogs with DM treated with curcumin had a significantly longer survival time than those who did not receive this treatment [97]. Daily physical therapy can be implemented to decrease the progression of the clinical signs of the disease [97,98,102]. The median survival time for dogs with DM ranges from 10 to 36 months [83,97]. Given the progressive, fatal nature of DM, humane euthanasia is most often pursued before the end-stage of the disease.

### 3.3. Spinal Cord Neoplasia

Spinal cord tumours, as with brain neoplasms, are more commonly diagnosed in older dogs [103,104,105,106,107,108,109,110]. There are different classification schemes for spinal cord neoplasia. One of them is based on their location in transverse sectioning relative to the spinal cord and dura, and they are classified as intramedullary, intradural extramedullary, or extradural [103,104,110,111]. Intramedullary tumours are uncommon, with a reported distribution of approximately 15%. Primary intramedullary spinal cord neoplasms (gliomas and nephroblastoma) are more common than secondary tumours (metastases) [103,106,111]. Intradural extramedullary neoplasia makes up approximately 35% of spinal cord neoplasia and includes mainly meningiomas and nerve sheath neoplasms [103,104,105]. Finally, extradural tumours are the most common spinal neoplasia in dogs, with a reported distribution of approximately 50%. The most prevalent neoplasms in this group are primary musculoskeletal, round cell neoplasia, and metastases [103,105,107,108,109]. 

Spinal cord neoplasms most commonly present in large-breed dogs older than 5 years [103,104,105,110]. They tend to cause chronic progressive neurological deficits, although some cases can show acute deterioration due to pathological fracture of the vertebrae secondary to bone tumours [103,107]. Presenting clinical signs can range from pain as a solitary sign to lameness, paresis, or even paralysis, associated most frequently with the spinal cord segment T3-L3 [109,111]. Pain is a commonly presenting sign with extradural or intradural extramedullary lesions, but it is also frequent in intramedullary neoplasia [103,104,107,111]. 

**Figure 4:**
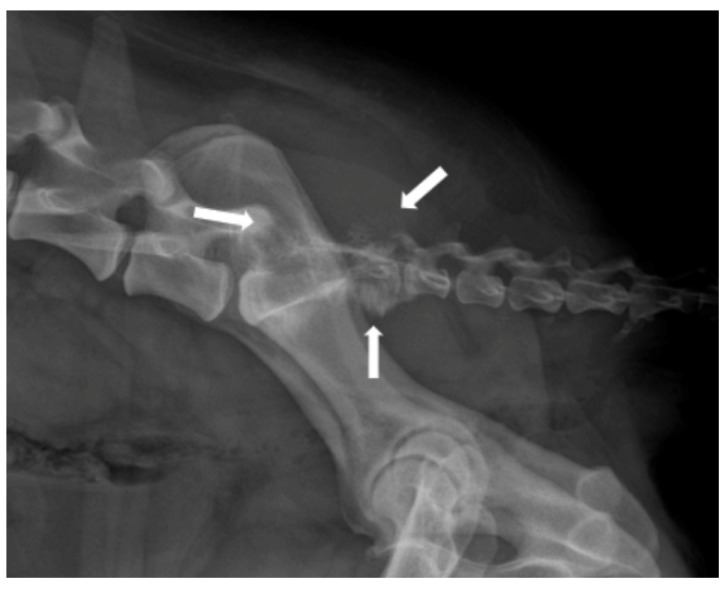
Lateral radiographic view of a sacrum osteosarcoma. Notice the severe osteolysis of the vertebral body and arch of the sacrum (arrows).

MRI is the method of choice for visualizing spinal tumours. Many types of bone neoplasms, such as multiple myeloma and vertebral osteosarcoma, may be evident on plain films as osteolytic/osteoproliferative processes and pathologic fractures (Figure 4). Occasionally, a pressure-enlarged intervertebral foramen will be noted from a nerve root tumour exiting the spinal canal [103,112].

**Figure 5:**
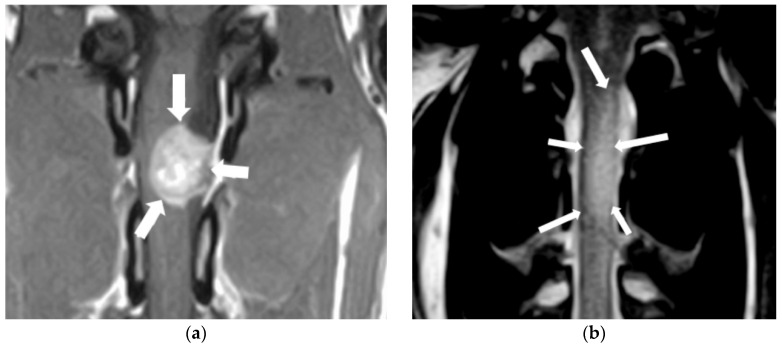
MRI images of a spinal cord nerve sheath tumour (**a**) and an oligodendroglioma (**b**). (**a**) Dorsal post-contrast T1 image. Note a strongly contrast-enhancing extradural mass at the level of the left C2-C3 intervertebral foramen causing a severe spinal cord compression (arrows). (**b**) Dorsal T2-weighted image showing a diffuse, hyperintense intramedullary lesion over the body of vertebrae C2 and C3, with a left-side lateralization (arrows).

However, most patients with spinal cord neoplasms will require an MRI to establish the relationship of the tumour with the dura mater, involved structures, the degree of spinal cord compression, and changes in spinal cord parenchyma (Figure 5) [103,107,108,109,111,113]. 

Moreover, several MRI features has been reported to try to identify different types of spinal cord extradural neoplasia [107,108,109]. However, the classification of lesions on MRI as intradural extramedullary, intramedullary, or both should be treated with caution. Differences of opinion exist among specialists, failure to designate the correct classification is common, and biopsy should be performed to confirm the presumptive diagnosis [113].

The treatment options for spinal neoplasia include supportive therapy, surgical removal, chemotherapy, and radiation therapy. As it was described in brain tumours, supportive therapy focuses on pain management and the reduction of associated oedema and inflammation with the use of glucocorticoids and analgesics [103,112]. Surgical removal is most often reserved for extramedullary neoplasia because most intramedullary neoplasia are difficult to remove without damaging the surrounding parenchyma [105,110,114]. Radiation therapy can be used as a primary therapy when surgery is not feasible, as an adjunctive therapy for lymphoma and incompletely excised tumours, and to provide the palliative relief of pain associated with osteolysis or nerve compression [110,115]. Chemotherapy is almost always reserved for use in cases of lymphoma and bone neoplasia [103,111]. Prognosis is dependent on the location and tissue type of the tumour, as well as the severity of clinical signs. Overall, prognosis is fair to grave, especially for intramedullary neoplasms that cannot be surgically excised [103,105,110,111].

A summary of the main data on the clinical presentation, diagnosis, and treatment of spinal cord diseases is included as Table S2 in the Supplementary Materials.

## 4. Conclusions

Improving the care and feeding of dogs will increase their life expectancy. Neurological diseases are common in geriatric dogs, and although they can present with dramatic clinical signs, in a high percentage of cases, adequate management and a good quality of life for the patient can be achieved with symptomatic treatment. Research being conducted in the diagnosis and treatment of more severe conditions, such as tumours or degenerative myelopathy, will help improve the survival and quality of life of sick dogs and their caregivers.

## Data Availability

No new data were created or analyzed in this study. Data sharing is not applicable to this article.

## Supplementary Materials

The following supporting information can be downloaded at: https://www.mdpi.com/article/10.3390/ani14121753/s1, Table S1: Summary of main data on clinical presentation, diagnosis, and treatment of brain diseases; Table S2: Summary of main data on clinical presentation, diagnosis, and treatment of spinal cord diseases.
