# Supplementary material for: Common Neurologic Diseases in Geriatric Dogs
Source: Animals (Basel). 2024 Jun 10;14(12):1753. doi: 10.3390/ani14121753 (PMC11200570; doi:10.3390/ani14121753)
Supplement: Supplementary file 1 [file animals-14-01753-s001.zip › animals-3043763-supplementary.pdf]

Table S1: Summary of main data on clinical presentation, diagnosis, and treatment of brain diseases.

DISEASE	Clinical presentation	Diagnosis	Treatment
Brain neoplasia	Behavioural changes<br>Seizures<br>Neurological deficits of a focal brain lesion	CBC, biochemistry, urinalysis<br>Thoracic radiography, abdominal US<br>MRI: test of choice<br>CSF: nonspecific findings<br>Biopsy: definitive diagnosis	Symptomatic: corticosteroids and antiepileptics. (MST: 65 days)<br>Chemotherapy: glioma (lomustine, carmustine, temozolamide), meningioma (hydroyurea)<br>Surgery: meningiomas (MST: 386 days), gliomas (technically demanding)<br>Radiation therapy: increase survival and seizure freedom
Cerebrovascular disease	Acute neurological signs of a focal brain, thalamic or cerebellar lesion	CBC, biochemistry, urinalysis<br>Blood pressure measurement,<br>Thyroid, and adrenal function<br>MRI: test of choice<br>CT: sensitive for acute haemorrhage<br>CSF: nonspecific findings	Symptomatic: fluid and oxygen therapy<br>Etiologic: if an underlying cause was identified<br>Fibrinolytic therapy: not evaluated
Idiopathic vestibular syndrome	Acute vestibular signs<br>Nystagmus<br>Facial neve paralysis: common<br>Absence of Horner's syndrome	CBC, biochemistry, urinalysis<br>Blood pressure measurement,<br>Thyroid function<br>Otoscopic examination<br>MRI/CT: absence of improvement with symptomatic therapy	Symptomatic: fluid therapy, antiemetics (maropitant, ondansetron).<br>Betahistidine?<br>Physiotherapy?

Table S2: Summary of main data on clinical presentation, diagnosis, and treatment of spinal cord diseases.

DISEASE	Clinical presentation	Diagnosis	Treatment
Intervertebral disc protrusion	Chronic, progressive myelopathy<br>Pain: nerve root compression	Radiography: nonspecific findings<br>MRI: test of choice	Medical: relative rest, analgesic, anti-inflammatory drugs, physiotherapy<br>Surgery: lateral corpectomy, hemilaminectomy and annulectomy<br>Others: perineural glucocorticoid injection, medical ozone
Degenerative myelopathy	Chronic, progressive upper neuron myelopathy of the pelvic limbs but that progress to the thoracic limbs and brainstem<br>Not painful	MRI: discharge other myelopathies<br>CSF: absence of anomalies<br>SOD1<br>Definitive: postmortem	Physiotherapy<br>Curcumin?
Spinal cord neoplasia	Chronic, progressive myelopathy (preference T3-L3)<br>Pain	Radiography: osteolytic lesions bone tumours, pathological fractures<br>MRI: test of choice. Classification of the lesion: extramedullary, intradural-extramedullary, intramedullary	Symptomatic: analgesics and anti-inflammatory drugs<br>Surgery: difficult in intramedullary tumours<br>Chemotherapy: lymphoma and bone neoplasia<br>Radiation therapy
